# D609 inhibition of phosphatidylcholine-specific phospholipase C attenuates prolonged insulin stimulation-mediated GLUT4 downregulation in 3T3-L1 adipocytes

**DOI:** 10.1007/s13105-022-00872-x

**Published:** 2022-01-20

**Authors:** Jinhui Ma, Xu Zhang, Yankun Song, Yan Qin, Yinghui Tan, Lishuang Zheng, Baoqian Cheng, Xin Xi

**Affiliations:** 1 Department of Endocrinology, Affiliated Hospital of Hebei University, Baoding, 071000 China; 2 Baoding Maternal and Child Hospital, Baoding, 071000 China; 3 School of Medicine, Hebei University, Baoding, 071000 China; 4 Central Laboratory, Affiliated Hospital of Hebei University, Baoding, 071000 China; 5 School of Medicine, Hebei Medical University, Shijiazhuang, 050017 China

**Keywords:** Phosphatidylcholine-specific phospholipase C, Glucose transporter 4, Insulin stimulation, 3T3-L1 adipocytes

## Abstract

Glucose uptake is stimulated by insulin via stimulation of glucose transporter 4 (GLUT4) translocation to the plasma membrane from intracellular compartments in adipose tissue and muscles. Insulin stimulation for prolonged periods depletes GLUT4 protein, particularly in highly insulin-responsive GLUT4 storage vesicles. This depletion mainly occurs via H_2_O_2_-mediated retromer inhibition. However, the post-receptor mechanism of insulin activation of oxidative stress remains unknown. Here, we show that phosphatidylcholine-specific phospholipase C (PC-PLC) plays an important role in insulin-mediated downregulation of GLUT4. In the study, 3T3-L1 adipocytes were exposed to a PC-PLC inhibitor, tricyclodecan-9-yl-xanthogenate (D609), for 30 min prior to the stimulation with 500 nM insulin for 4 h, weakening the depletion of GLUT4. D609 also prevents insulin-driven H_2_O_2_ generation in 3T3-L1 adipocytes. Exogenous PC-PLC and its product, phosphocholine (PCho), also caused GLUT4 depletion and promoted H_2_O_2_ generation in 3T3-L1 adipocytes. Furthermore, insulin-mediated the increase in the cellular membrane PC-PLC activity was observed in Amplex Red assays. These results suggested that PC-PLC plays an important role in insulin-mediated downregulation of GLUT4 and that PCho may serve as a signaling molecule.

## Introduction

Glucose transporter 4 (GLUT4) is a primary glucose transporter in the muscle and adipose tissue that plays an important role in the regulation of blood glucose homeostasis. It is inserted into the budding GLUT4 storage vesicles (GSVs) that are sequestered within cells [13]. The blood glucose increase after meals promotes insulin secretion, which induces GSVs to translocate to the plasma membrane by exocytosis. Then, the GLUT4 inserted in the plasma membrane transports glucose into the cells [4, 9, 11]. After the disappearance of the insulin signal, cell surface GLUT4 is recovered from the extracellular membrane by endocytosis and separated from the circulation between the endosomes and plasma membrane through the sorting process, resulting in the formation of GLUT4 vesicles or the occurrence of GLUT4 transport to the trans-Golgi network through the endosome [3, 12, 37]. The balance of GLUT4 translocation, exocytosis, and endocytosis is important for glucose homeostasis [10]. Prolonged insulin stimulation causes a reduction in GLUT4 [7, 8, 23]. This depletion is selectively from the GSVs and is thus accompanied by a lack of response to insulin by the glucose transport system [15, 19]. Enhanced GLUT4 degradation in lysosomes is primarily responsible for this depletion; previous studies have demonstrated that GLUT4 protein turnover is accelerated about three-fold with insulin in 3T3-L1 adipocytes [23] although the precise mechanism is not fully understood. Previous research has also revealed a unique oxidative stress-mediated insulin signal cascade that regulates the fate of GLUT4 by interfering with the function of the retromer complex. Insulin leads to the disassembly of the retromer complex from low-density microsomal (LDM) membranes, causing the GLUT4 sorting at endosomes to switch from recycling to the trans-Golgi network to lysosomal degradation. The signaling mechanism of this insulin action is unique in that it depends on insulin-generated oxidative stress, particularly via hydrogen peroxide (H_2_O_2_), as well as on the activity of protein kinase CK2 but not phosphatidylinositol 3-kinase or extracellular signal-regulated kinase 1/2 (Erk1/2). In brief, insulin receptor tyrosine kinase activation stimulates H_2_O_2_ generation, which may increase the apparent activity of CK2. Moreover, CK2-induced vesicle protein sorting 35 (the cargo-selective subunit of retromer complex) phosphorylation may dissociate the retromer complex from the LDM membrane, causing the GLUT4 sorting direction to switch to lysosomes. This may cause a shortening of the GLUT4 half-life and depletion of GLUT4 in GSVs [18]. However, many intermediate steps remain to be clarified.

Tricyclodecan-9-yl-xanthogenate (D609) has antioxidant properties in many cell types [30, 33, 36] and scavenges H_2_O_2_ [14]. It is also a selective competitive inhibitor of phosphatidylcholine-specific phospholipase C (PC-PLC) which breaks down phosphatidylcholine (PC) to 1, 2-diacylglycerol (DG) and phosphocholine (PCho). Evidence has shown that PC-PLC plays a major role in the metabolism, growth, inflammation, differentiation, aging, and apoptosis of mammalian cells [6, 20, 27, 29, 31, 35]. In CHO cells, PC-PLC is a key molecule mediating the insulin-induced enhancement of human interleukin-6 expression, which strongly suggests that PC-PLC plays an important role in the insulin signaling network [34]. Insulin also induces the translocation of PC-PLC from a perinuclear cytoplasmic region to the plasma membrane in NIH-3T3 fibroblasts [22]. These observations have led us to investigate whether D609 could inhibit the prolonged insulin-generated oxidative stress that leads to GLUT4 depletion and whether PC-PLC is implicated in this insulin effect of GLUT4 in 3T3-L1 adipocytes. In the present study, inhibition of PC-PLC by D609 is shown to attenuate downregulation of GLUT4 by prolonged insulin treatment in 3T3-L1 adipocytes. Thus, this study reveals a unique PC-PLC-mediated insulin signal cascade is involved in the regulation of the fate of GLUT4.

## Materials and methods

### Materials and antibodies

Phosphocholine (HY-B2233), D609 (HY-70072), U-73122 (HY-13419), and GW4869 (HY-19363) were procured from MedChemExpress (Monmouth Junction, NJ), while 1, 2-dioctanoyl-sn-glycerol (800800O), 1-butanol (281,549), and hemicholinium-3 (H108) were procured from Sigma (St. Louis, MO). The Amplex Red Phosphatidylcholine-Specific Phospholipase C Assay Kit and PC-PLC (A12218) were obtained from Molecular Probes (Eugene, OR, USA); pHyPer-cyto (FP941) vector was procured from Evrogen (Moscow, Russia). Monoclonal antibodies against GLUT4 (2213S, 1:1000) were purchased from Cell Signaling (Danvers, MA, USA), and α-tubulin mouse monoclonal antibody (66,031–1-Ig, 1:10,000) was procured from Proteintech (Chicago, IL).

### Cell lines and cell culture

3T3-L1 (CL-173) cell line was procured from American type culture collection (Rockville, MD). The cells were grown in 10% calf serum-supplemented Dulbecco’s modified Eagle’s medium with 4.5 g/l d-glucose and maintained at 37 °C in 5% CO_2_. The differentiation of the cells into adipocytes was performed as described previously [28]. In brief, 2 days after reaching confluency, the cells were switched into a differentiation medium (DMEM supplemented with 1.5 g/l d-glucose and 10% fetal bovine serum, 115 µg/ml 3-isobutyl-1-methylxanthine, 1 µM dexamethasone, and 10 µg/ml insulin) and maintained for 2 days. The differentiation medium was then replaced with DMEM plus 1.5 g/l d-glucose supplemented with 10% FBS and 10 µg/ml insulin for another 2 days and maintained in DMEM containing 1.5 g/l d-glucose supplemented with 10% FBS. The adipocytes were used for experiments on days 8–10 after differentiation.

### Cell lysis and Western blotting

The homogenization of the cells was done in PBS with a cocktail of complete protease inhibitors (Roche, 05,892,791,001) via a Dounce tissue grinder, and the homogenates were centrifuged for 5 min at 1200* g* and 4 °C. The protein concentration in the supernatant was measured by a Bio-Rad protein assay according to the manufacturer’s protocol (Bio-Rad Laboratories, Inc.). Supernatant aliquots containing 20 µg total proteins were electrophoresed on a 10% SDS–polyacrylamide gel. Following SDS-PAGE, the proteins were transferred to Immobilon-P membranes. Subsequently, the membranes were blocked with 10% (w/v) skimmed milk in Tris-buffered saline supplemented with 0.05% Tween 20 (TBS-T) for 1 h at room temperature before being incubated overnight with the indicated primary antibodies at 4 °C. After being washed with TBS-T, the membranes were incubated with horseradish peroxidase–conjugated goat anti-mouse or goat anti-rabbit secondary antibodies for 1 h at room temperature. The blots were visualized using Immobilion Western reagents (Merck Millipore, Billerica, MA, USA) according to the manufacture’s instruction. Densitometry analyses of the protein bands were conducted employing the ImageJ software.

### Total membrane protein extraction

The total membrane protein was extracted by an ExKine™ Total Membrane Protein Extraction Kit (KTP3004; Abbkine, Wuhan, China) according to the manufacturer’s instructions. Briefly, the cells were washed with ice-cold PBS and then homogenized in ice-cold extraction buffer with a Dounce homogenizer. After the cell suspension was incubated on ice for 10 min, the cell lysate was centrifuged at 10,000* g* for 5 min at 4 °C. The supernatant was incubated at 37 °C for 5 min. During the incubation, the tube was inverted once to mix the supernatant. Then, it was centrifuged at room temperature at 3000* g* for 3 min. The lower hydrophobic phase was greatly enriched with hydrophobic and raft-associated proteins. After the supernatant was washed with Wash Buffer twice to remove residual hydrophilic proteins, the hydrophobic phase was immediately applied for the downstream assay.

### *In vitro* PC-PLC activity assay

The PC-PLC activity was assayed in vitro via the Amplex Red phosphatidylcholine-specific phospholipase C assay kit (A12218; Molecular Probe, Eugene, OR, USA) according to the manufacturer’s protocol (modified as described [26]). The fluorescence was measured in a fluorescence microplate reader (Fluoroskan Ascent™ FL, Thermo Scientific, Waltham, MA, USA) using excitation of 544 nm and emission detection at 590 nm. The resorufin fluorescence at 0 min was normalized to100%.

### Determination of cytosolic H_2_O_2_

Electroporation was used to transfect 3T3-L1 adipocytes with 30 µg of the pHyPer-Cyto plasmid vector (Evrogen, Moscow, Russia). The transfected cells were seeded in 35-mm glass-bottom culture dishes. Following 24 h of incubation, the culture medium was replaced with Hanks’ Balanced Salt Solution containing 138 mM NaCl, 5.4 mM KCl, 1.3 mM CaCl_2_, 0.5 mM MgCl_2_, 0.38 mM MgSO_4_, 0.44 mM KH_2_PO_4_, 0.34 mM Na_2_HPO_4_, 5.5 mM d-glucose, and 20 mM Hepes/NaOH (pH 7.4). To monitor the H_2_O_2_, the cells were excited with a light beam at a 440-nm wavelength, and emission images were captured at 1-min intervals via an AQUACOSMOS/ASHURA fluorescence imaging system (Hamamatsu Photonics, Hamamatsu, Japan).

### Statistics

The statistical analyses were performed using GraphPad Prism 5 (GraphPad Software, USA). The data were analyzed by Student’s *t*-test to compare the differences between the groups, and *p* < 0.05 was considered statistically significant.

## Results

### Prolonged insulin stimulation depends on PC-PLC activity to downregulate GLUT4

**Fig. 1:**
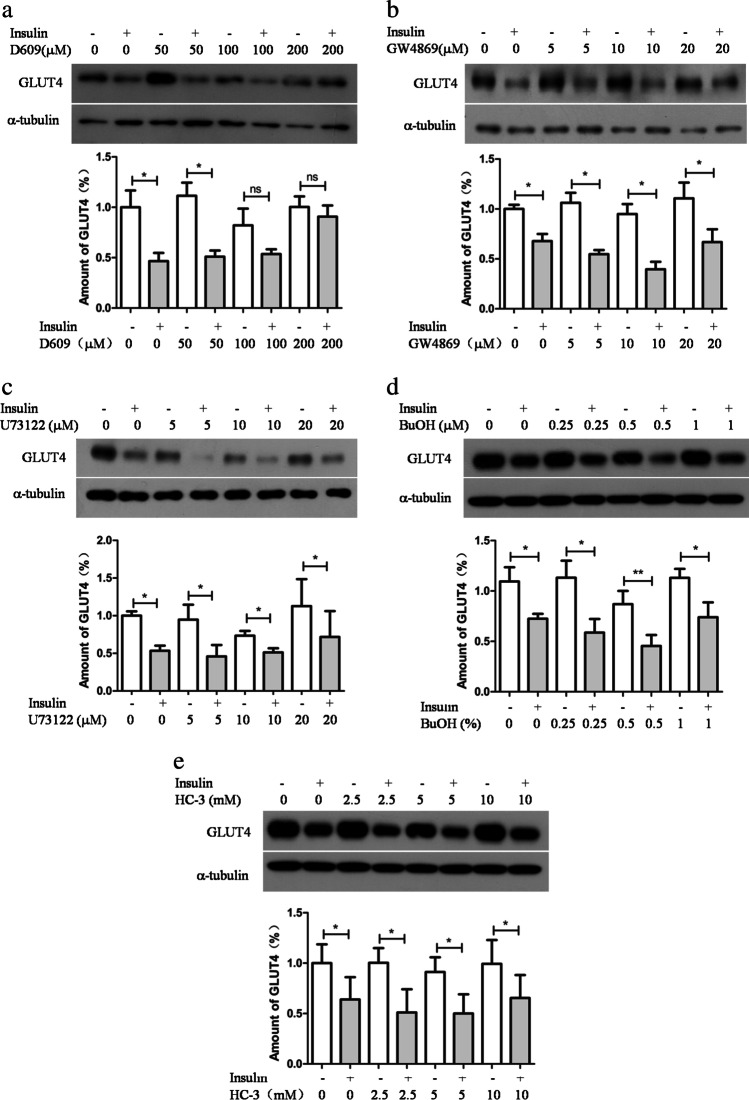
Prolonged insulin stimulation requires PC-PLC activity for the downregulation of GLUT4. Prior to stimulation with insulin (500 nM) for 4 h, the 3T3-L1 adipocytes were treated with or without the following compounds at indicated concentrations for 30 min: D609 (**a**), GW4869 (**b**), U73122 (**c**), 1-Butanol (**d**), or HC-3 (**e**). GLUT4 were detected by immunoblotting. The upper panels show the representative immunoblots for GLUT4, and the lower panels were used for quantifying the GLUT4. The results shown are the mean ± *SD* (*n* = 3)

In the current study, 3T3-L1 adipocytes were exposed to different doses of D609 for 30 min prior to being stimulated with 500 nM insulin for 4 h. The immunoblotting results showed that the cells stimulated with insulin alone had a deficit of GLUT4. Whereas, the cells exposed to 100 and 200 nM of D609 before insulin stimulation had a weakened GLUT4 deficit (Fig. 1a), D609 is a selective competitive inhibitor of PC-PLC [1], which conducts the hydrolysis of PC to PCho and DG. Additionally, PCho can be produced by neutral sphingomyelinase from sphingomyelin. However, GW4869 inhibition of sphingomyelinase does not prevent insulin downregulation of GLUT4 (Fig. 1b); DG can also be produced from phosphatidylinositol by phosphoinositide-specific phospholipase C (PI-PLC). An inhibitor of PI-PLC, U73122, does not prevent insulin downregulation of GLUT4 (Fig. 1c); phospholipase D (PLD) also hydrolyzes PC to form phosphatidic acid, which is then dephosphorylated to DG by phosphatidic phosphatase. The specific inhibitor of PLD, 1-butanol, does not prevent insulin downregulation of GLUT4 either (Fig. 1d); choline kinase phosphorylation of choline also produces PCho; the choline kinase inhibitor, hemicholinium-3 (HC-3), does not prevent insulin downregulation of GLUT4 (Fig. 1e).

### PC-PLC downregulates GLUT4 concentrations in 3T3-L1 adipocytes

**Fig. 2:**
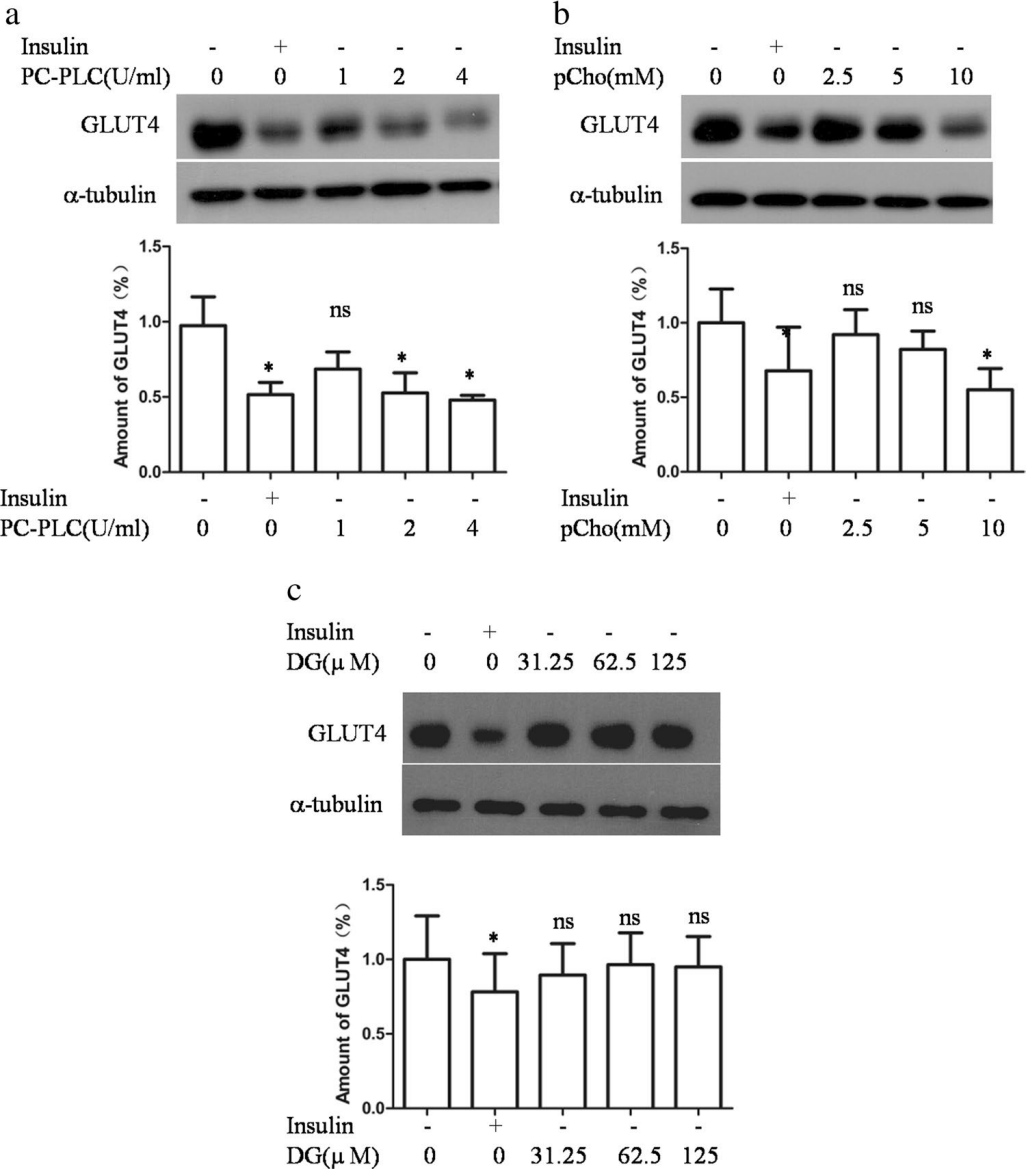
Effect of exogenous PC-PLC and its products (PCho and DG) on GLUT4 concentrations. The 3T3-L1 adipocytes were treated with or without the following at indicated concentrations for 4 h: PC-PLC (**a**), PCho (**b**), and DG (**c**). After incubating, the cells were lysed and processed for immunoblotting. The upper panels show the representative GLUT4 immunoblots, and the lower panels display the GLUT4 quantification. The results shown are the mean ± *SD* (*n* = 3)

Next, exogenous PC-PLC downregulation of GLUT4 concentration was explored. The 3T3-L1 adipocytes were exposed to exogenous PC-PLC at the indicated doses (Fig. 2a). The GLUT4 protein decreased in a dose-dependent manner, and 2 U/ml PC-PLC had a similar effect on the GLUT4 concentration as prolonged insulin stimulation. Furthermore, a product of PC-PLC, PCho, downregulated GLUT4 (Fig. 2b), but the other product, DG, showed no obvious effect (Fig. 2c).

### Insulin activates PC-PLC in 3T3-L1 adipocytes

**Fig. 3:**
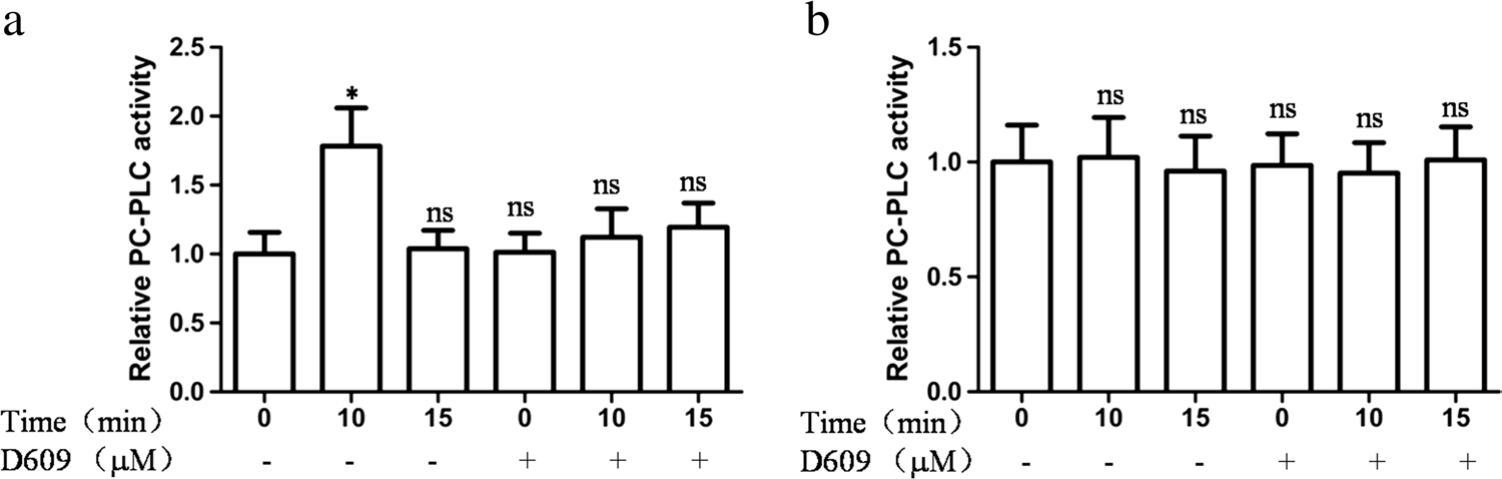
Effect of insulin stimulation on PC-PLC activity. The 3T3-L1 adipocytes were treated with or without D609 (200 μM) for 30 min and then stimulated with insulin for indicated time intervals. The PC-PLC activity in the membranes (**a**) and total lysate (**b**) were assayed as described in the “Materials and methods” section

Amplex Red assay is often used for measuring PC-PLC activity [1, 26]. To analyze the insulin activation of PC-PLC, 3T3-L1 adipocytes were incubated with insulin for different lengths of time. Amplex Red assays for total membrane proteins showed a 1.78% ± 0.27% increase of resorufin fluorescence (and, therefore, PC-PLC activity) after insulin stimulation for 10 min (Fig. 3a). The resorufin fluorescence dropped to control levels at 15 min. When the cells were pre-treated with D609 before insulin stimulation, the resorufin fluorescence levels changed minimally, confirming the inhibitory effect on PC-PLC activity [2]. The total cell lysates showed no significant change in the resorufin fluorescence after the insulin stimulation (Fig. 3b). Thus, the effects of insulin stimulation are dependent on the PC-PLC activity associated with cellular membranes.

### PC-PLC increases H_2_O_2_ in 3T3-L1 adipocytes

**Fig. 4:**
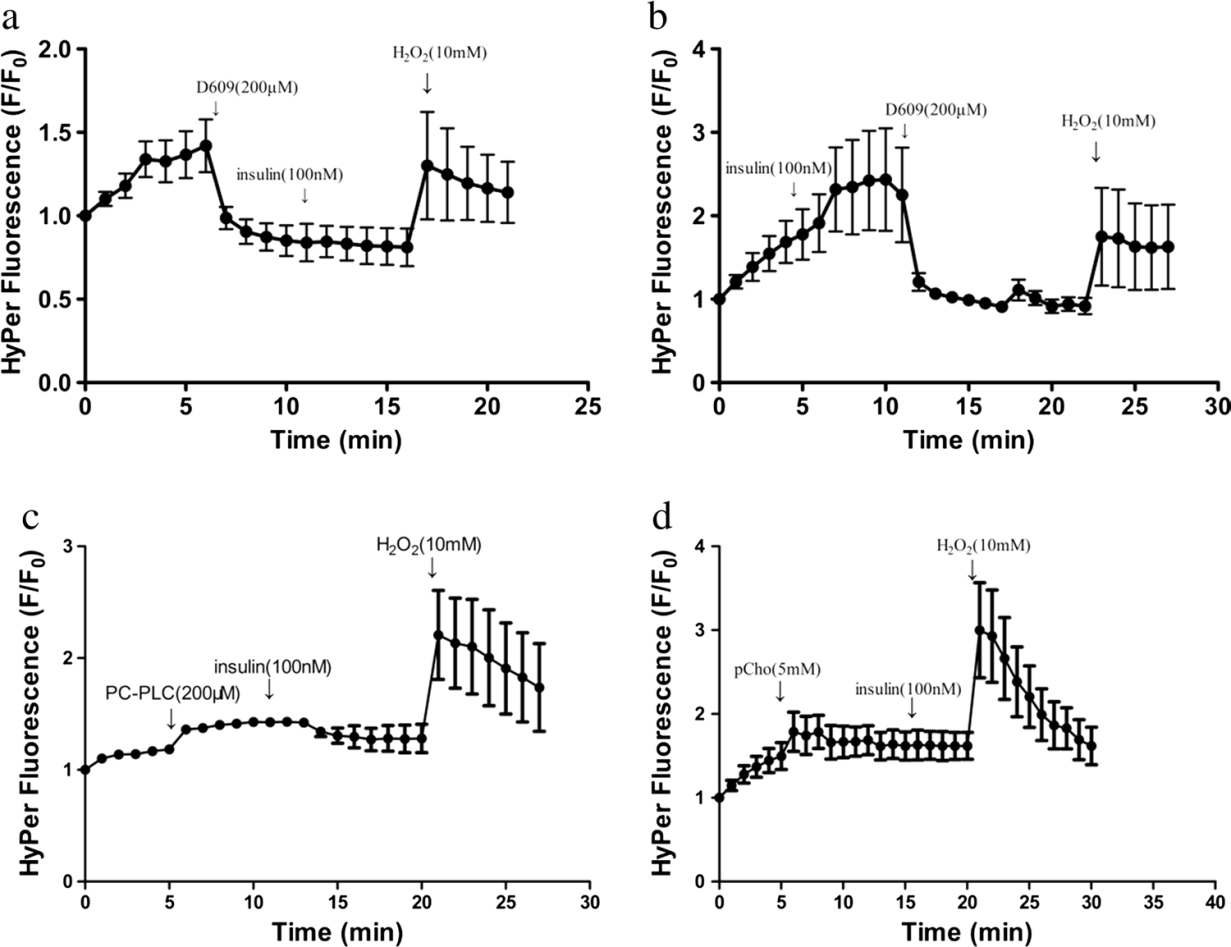
Effect of PC-PLC on H_2_O_2_. Transfection of the 3T3-L1 adipocytes was performed by electroporation with the pHyPer-Cyto expression vector (30 μg), after which the cells were cultured for 24 h on a 35-mm glass dish prior to the fluorescence measurements. The cells were stimulated with indicated concentrations of insulin followed by D609 (**a**), insulin (**b**), PC-PLC (**c**), or PCho (**d**) at the time points indicated with arrows. The data are presented as the mean ± *S.E.* (*n* = 5)

Insulin promotes the production of the reactive oxygen species, primarily H_2_O_2_, that regulate GLUT4 concentrations [18]. Thus, an investigation of the role of D609 and PC-PLC in the generation of H_2_O_2_ was conducted. Fluorescent protein, pHyPer, which is sensitive to H_2_O_2_, was used to monitor the production of H_2_O_2_. A previous study indicated that insulin elicits a rapid and sustained (over 150 min) elevation of the pHyPer fluorescence intensity [18]. Here, the pHyPer fluorescence intensity decreased after the addition of D609 (Fig. 4a). Furthermore, pretreatment with D609 before the insulin stimulation prevented an increase in the pHyPer fluorescence intensity (Fig. 4b), while both PC-PLC and PCho elicited an increase in the pHyPer fluorescence intensity (Fig. 4c, d).

## Discussion

In the current study, the mechanism of GLUT4 downregulation by chronic insulin stimulation was investigated, and the results provided evidence that PC-PLC is essential for the regulation of GLUT4 concentration. This finding was supported by several observations: First, pretreatment of 3T3-L1 adipocytes with D609, an inhibitor of PC-PLC, attenuated the downregulation of GLUT4 protein levels by insulin stimulation; D609 has also been reported to upregulate PLD activity [25, 32] or block sphingomyelin synthase [17]. However, specific inhibition of PLD by BuOH and sphingomyelin by GW4869 does not attenuate the downregulation of GLUT4 by prolonged insulin stimulation. Second, exogenous PC-PLC and its reaction product, PCho, downregulated the GLUT4 concentrations in the 3T3-L1 adipocytes, suggesting an essential role of PC-PLC in the downregulation of GLUT4. Additionally, PCho may act as a signaling molecule for this effect. Third, insulin activated the membrane PC-PLC in 3T3-L1 adipocytes. The Amplex Red assays showed an increase in the PC-PLC activity after insulin stimulation in the total membrane proteins but not the total cell lysates. These findings were consistent with previous reports that insulin induces PC-PLC migration from the perinuclear cytoplasm to the plasma membrane in NIH-3T3 fibroblasts [22]. Similar PC-PLC expression has also been reported on the outer membrane of human NK cells. The amount of externalized PC-PLC is elevated two-fold after the activation of cells with cytokines [21]. Such observations support the membrane translocation of PC-PLC, which may be in an optimal location to break down the PC present on the exterior membrane surface. As PC-PLC is positioned downstream of Ras [5] and Ras proteins are activated by insulin, activation of PC-PLC might occur concomitantly. However, the precise mechanisms remain to be elucidated. When the adipocytes were pre-treated with D609 before insulin stimulation, the resorufin fluorescence did not significantly change, confirming D609’s inhibitory effect on the activity of PC-PLC.

Previous studies have demonstrated that insulin promotes the production of reactive oxygen, mostly H_2_O_2_, which regulates GLUT4 concentrations [18]. Here, real-time measurement of pHyPer fluorescence demonstrated that insulin stimulates the rapid and continuous formation of H_2_O_2_, and this effect was blocked by D609. Moreover, exogenous PC-PLC and its product PCho also promoted H_2_O_2_ production. These results suggested that PC-PLC occupies a key position in signal transduction from insulin to oxidative stress in 3T3-L1 adipocytes. They were in agreement with those of earlier reports indicating that PC-PLC activity is regulated in a redox-dependent manner [16] and implicate in the NADPH oxidase cascade that regulates cigarette smoke extract induce heme oxygenase-1 expression in mouse brain endothelial cells [24]. A recent study reported that in the retinal pigmented epithelium, D609 achieves its antioxidant effects primarily through elevating the expression of the metallothionein (MT) family, which is well known for its ability to eliminate oxidative risk factors [33]. In our study, the real-time measurement results of pHyPer fluorescence also show that D609 blocked insulin-generated oxidative stress in 3T3-L1 adipocytes. Meanwhile, PC-PLC and PCho elevated the oxidative stress, and D609’s inhibitory effect on the activity of PC-PLC was also confirmed by the Amplex Red assays, thus suggesting that PC-PLC may implicate in attenuation of prolonged insulin stimulation-generated oxidative stress. The antioxidative mechanism of D609 may differ in different cell types. However, whether the MT family participates in this insulin action has not been investigated. Further work is necessary to elucidate the precise mechanism of D609 and PC-PLC on this insulin effect in 3T3-L1 adipocytes.

In conclusion, inhibition of PC-PLC by D609 attenuates insulin-driven GLUT4 depletion and H_2_O_2_ generation. Exogenous PC-PLC and its product, phosphocholine (PCho), also caused GLUT4 depletion and promoted H_2_O_2_ generation. Furthermore, an insulin-mediated increase in the cellular membrane PC-PLC activity was observed in Amplex Red assays. These results suggested that PC-PLC plays an important role in insulin-mediated downregulation of GLUT4 and that PCho may serve as a signaling molecule.

## Data Availability

The data that support the findings of this study are available from the corresponding author upon reasonable request.
